# β-Hydroxy-β-Methylbutyric Acid Promotes Repair of Sheep Myoblast Injury by Inhibiting IL-17/NF-κB Signaling

**DOI:** 10.3390/ijms24010444

**Published:** 2022-12-27

**Authors:** Juan Zheng, Bo Li, Yiting Yan, Xiaoyu Huang, Enping Zhang

**Affiliations:** College of Animal Science and Technology, Northwest A&F University, Xianyang 712100, China

**Keywords:** myoblast, apoptosis, proliferation, HMB, sheep

## Abstract

Delayed muscle development and impaired tissue repair are common occurrences in sheep reared for mutton. Therefore, understanding the regulatory mechanisms involved in muscle growth and development is critical for animal production. Skeletal muscle satellite cells (SMSCs) can simulate the proliferation and differentiation of muscle cells and could be induced to differentiate into myoblasts. β-hydroxy-β-methylbutyric acid (HMB) is an additive commonly used in animal production. This study examined the effect of HMB on myoblast injury repair using flow cytometry, EdU assay, RNA sequencing, Western blot, and ELISA. Our results showed that HMB could inhibit IL-17 expression and, in turn, inhibit NF-κB signaling. By acting on the downstream genes of NF-κB pathway IL-6, TNF-α and IL-1β, HMB inhibits the apoptosis and promotes the proliferation of myoblasts. The findings of this study provide insight into the mechanism by which HMB mediates myoblast injury repair in sheep.

## 1. Introduction

Tan sheep are a premium local sheep breed mainly kept for mutton. Skeletomuscular development directly impacts meat production performance [1]. With the increasing demand for animal meat products [2], effective muscle tissue development and repair of injured muscle tissues can improve the quality of meat products [3]. Muscle tissues are important in regulating metabolism and maintaining internal homeostasis. Both skeletal muscle satellite cells (SMSCs) and cytokines can directly or indirectly modulate tissue remodeling following skeletal muscle injury [4]. Excessive injury or impaired regeneration can lead to a significant decline or loss of muscle functions [5]. Thus, understanding the mechanisms of skeletal muscle tissue injury and repair could provide ideas for promoting muscle tissue development.

SMSCs are myogenic stem cells in the sarcolemma and basal lamina of skeletal muscle fibers [6]. SMSCs are named based on their anatomical relationship with muscle fibers and are very important for skeletal muscle growth and regeneration [7]. During animal growth and development, SMSCs are usually in a resting state and form a potential myogenic reservoir. External stimuli such as weight and trauma rapidly activate SMSCs to express the myogenic regulators *Myf5*, *MyoD*, *Myogenin*, and *MRF4* [8,9]. Finally, SMSCs undergo a series of complex myogenic processes which restore skeletal muscle structure and functions [10]. Therefore, the mechanisms of action of SMSCs in skeletal muscle formation and development have become an active topic of research.

β-hydroxy-β-methylbutyric acid (HMB) is a dietary supplement that has been linked with metabolic disorders [11,12]. Although these studies are still in their infancy and have not been deeply evaluated, they still provide informative findings. Studies have investigated the effect of HMB on quality of carcass, immune functions, morbidity rate, and mortality rate in animals [13]. In sows at the peak of their lactation, HMB supplementation increased colostrum production but decreased piglet growth [14]. To lower mortality in this patient population, HMB may be a significant treatment for respiratory muscle weakness brought on by infection in mice [15]. By increasing marbling score and lowering fat thickness, HMB esterification has the potential to enhance carcass quality. These adjustments may raise the quality rating [16]. It was also shown that HMB can boost immune performance, rate of growth, and muscle development [17]. HMB supplementation significantly enhances feed utilization in animals. HMB supplementation has recently gained popularity [18]. HMB calcium salts decrease serum creatine kinase (CK) and lactate dehydrogenase (LDH) levels in sedentary humans. In addition, HMB decreases muscle injury associated with resistance exercises, suggesting that HMB may promote muscle regeneration [19]. Although many studies have investigated the application of HMB in animal production, the molecular mechanisms of HMB effects remain unclear [20,21]. In the present study, we examined the role of HMB in muscle cell injury repair in Tan sheep.

## 2. Results

### 2.1. Immunofluorescence of SMSCs

**Figure 1:**
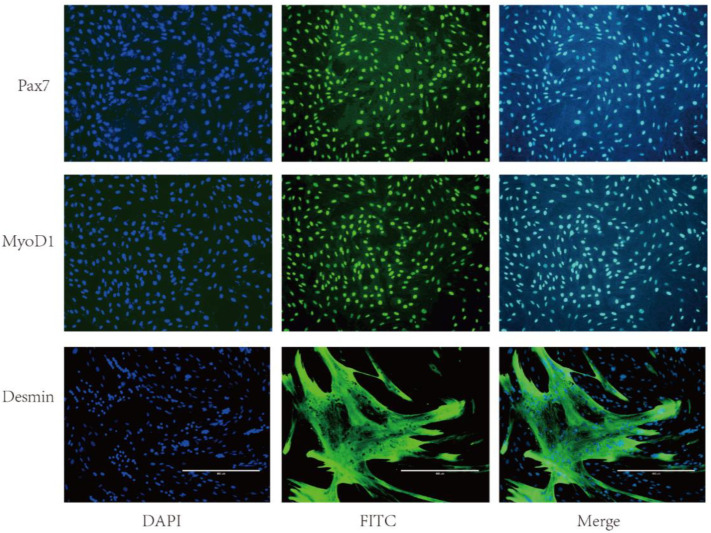
The expression of *Pax7*, *MyoD1*, and *Desmin* in SMSCs (scale: 1 bar represents 400 µm).

**Figure A1:**
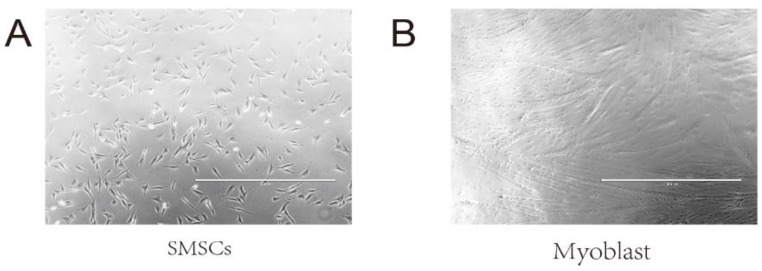
Myoblast differentiation. (**A**) SMSC morphology; (**B**) Myoblast morphology; (scale: 1 bar represents 400 µm).

SMSCs are flat cells with small surface protrusions (Figure A1A) and differentiate into spindle-shaped myoblasts (Figure A1B). Immunofluorescence (IF) staining showed that SMSCs were positive for *Pax7*, *MyoD1*, and *Desmin*. *Pax7* and *MyoD1* were expressed in the cell nuclei, while *Desmin* was expressed in the cytoplasm (Figure 1).

### 2.2. Cell Apoptosis and Proliferation

**Figure 2:**
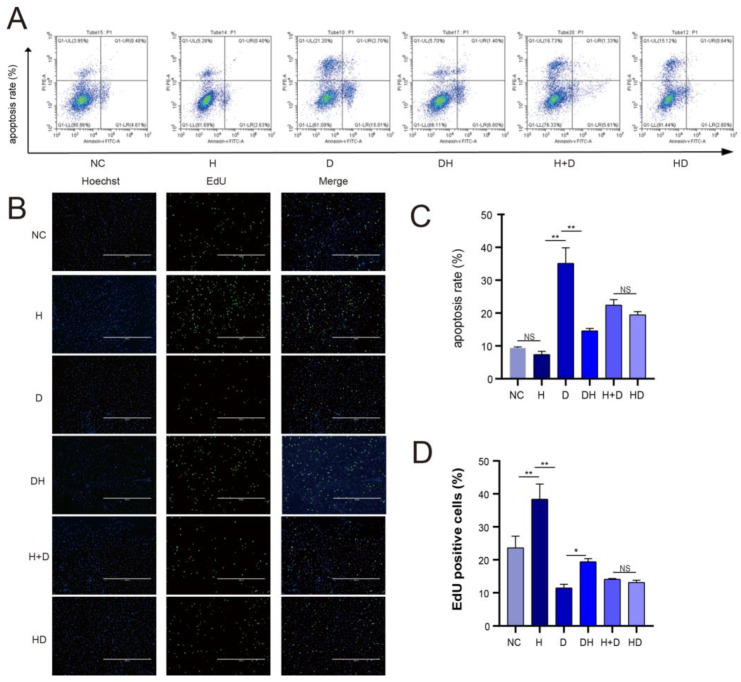
Effect of HMB on myoblast apoptosis and proliferation. (**A**) Flow cytometry plots; (**B**) EdU staining (scale: 400 µm); (**C**) Bar graph depicting cell apoptosis rate of each group; (**D**) Bar graph illustrating the frequency of EdU-positive cells in each group; *n* = 3, * *p* < 0.05, ** *p* < 0.01, NS (No Significant).

Flow cytometry results showed that the cell apoptosis rate was comparable between the H and NC (*p* > 0.05) but was dramatically higher in the D than in the H (*p* < 0.01) and the DH (*p* < 0.01). The cell apoptosis rate was also comparable between H+D and HD. These results demonstrated that HMB inhibited myoblast apoptosis (Figure 2A,C). The proliferation rate of cells in the six groups was also measured using the EdU assay. Cell proliferation was significantly decrease in the D than in the H (*p* < 0.01) and the DH (*p* < 0.05), indicating that HMB promoted myoblast proliferation (Figure 2B,D).

### 2.3. HMB Repairs Myoblast Injury by Blocking IL-17 and NF-κB Signaling

**Figure 3:**
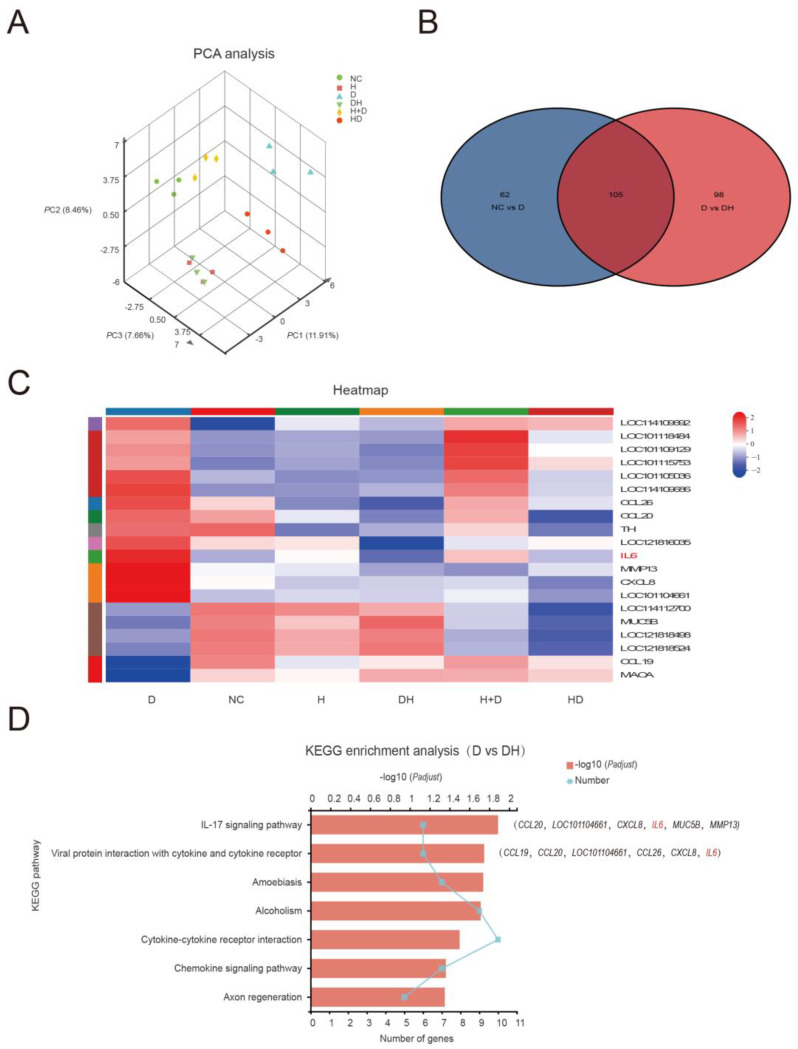
Transcriptomic analysis of the effect of HMB in the repair of myoblast injury. (**A**) PCA for the gene expression in myoblasts; (**B**) Venn diagram of NC, D and DH; (**C**) heatmap of top 20 differentially expressed genes; (**D**) KEGG enrichment analysis of the DEGs (D group vs. DH group; *n* = 3).

**Figure 4:**
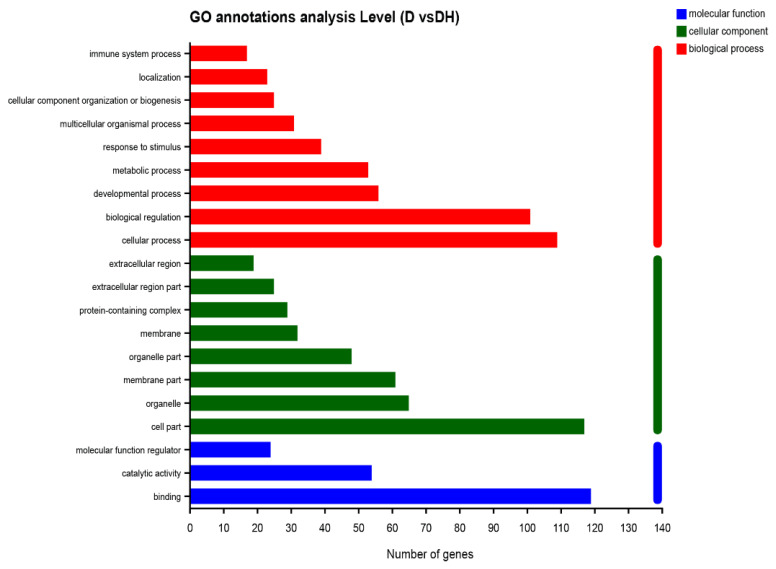
GO enrichment analysis with the differentially expressed genes of groups D and DH.

**Figure 5:**
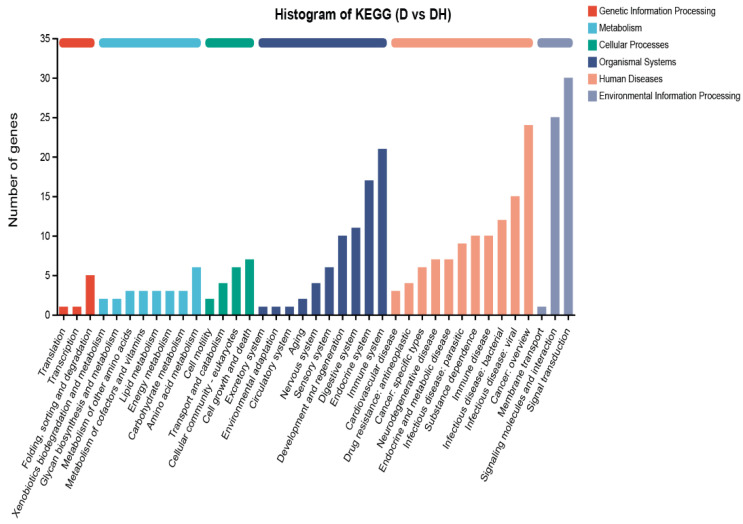
KEGG enrichment analysis with the differentially expressed genes of groups D and DH.

Principal component analysis (PCA) announced that the gene expression profiles between the NC and DH were similar, but there was a meaningful difference between the D group and other groups, with insignificant inter-sample variability (Figure 3A). The proliferation and apoptosis results revealed comparable findings. The NC, D, and DH groupings contain 105 differential genes, as illustrated in the Venn Chart (Figure 3B). In this study, 203 differential genes in the group D and DH groups were subjected to GO functional enrichment analysis. The results contained 45 GO items, which were split into three categories: molecular function (MF, 11), cellular component (CC, 15), and biological process (BP, 19), and are all depicted in Figure 4. The organismal systems and human diseases are the main subjects of the KEGG analysis’s histogram (Figure 5). Heatmap showed that HMB supplementation altered the gene expression in myoblasts. In particular, IL-6 expression was upregulated in the D but downregulated in the DH (Figure 3C).

**Figure 6:**
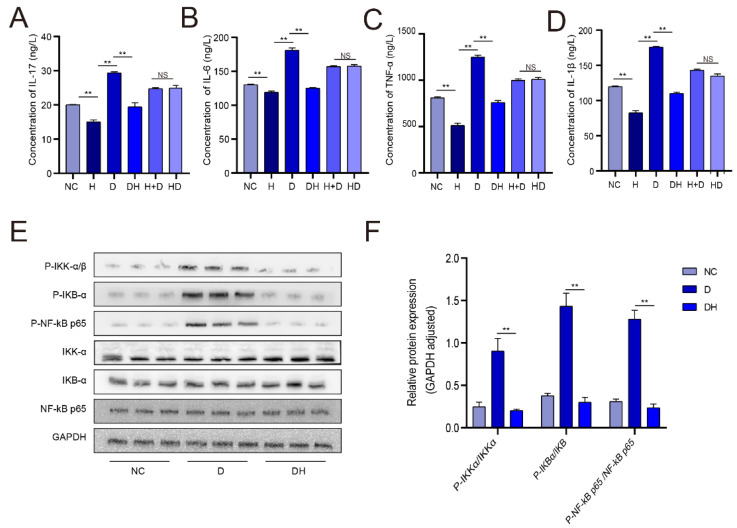
Validation of HMB-mediated repair of myoblast injury. (**A**) IL-17 expression measured by ELISA; (**B**) IL-6 protein expression measured by ELISA; (**C**) TNF-α protein expression measured by ELISA; (**D**) IL-1β protein expression measured by ELISA; (**E**,**F**) NF-κB p65, IKBα and IKKα protein expression measured by Western blot; *n* = 3, ** *p* < 0.01, NS (No Significant).

We found that the IL-17 gene was the major differentially expressed gene (DEG) that affected growth (Figure 3D). Therefore, we quantified IL-17 expression by ELISA. We found that IL-17 expression level was dramatically lower in the H than in the NC (*p* < 0.01), significantly higher (by about 1/3) in the D compared with the DH (*p* < 0.01), and comparable between DH and NC (Figure 6A). IL-17 signaling is primarily mediated via the nuclear factor (NF)-κB pathway. Western blot results indicated that the expression level of p-NF-κB p65, p-IKBα, and p-IKKα were significantly reduced in DH than in D (*p* < 0.01) (Figure 6E,F). IL-6 is the major target gene of the all groups. The expression level of IL-6 was significantly lower in H than in NC (*p* < 0.01), expressively decreased in DH compared with D (*p* < 0.01), but comparable between H+D and HD groups (*p* > 0.05) (Figure 6B). The trend in IL-6 protein expression level was generally consistent with that in IL-17 protein expression level. In addition, we also measured the expression level of tumor necrosis factor (TNF)-α and IL-1β protein levels. The results revealed that the trends in the expression of TNF-α and IL-1β were similar to those of IL-6 and IL-17 (Figure 6C,D).

## 3. Discussion

SMSCs are an important component of muscle tissues and have been used in muscle tissue studies [22]. *Pax7* is a common marker for resting SMSCs, while *MyoD1* and *Desmin* are key specific markers for the differentiation of SMSCs into myoblasts [23,24]. The identity of the extracted SMSCs was confirmed by detecting *Pax7*, *MyoD1*, and *Desmin*.

DEX-induced myoblast injury model was successfully established, as described by Yoshioka et al. Preliminary research revealed that 0.8 mmol/L DEX and 0.5 μmol/L HMB were the ideal level to cause myoblast damage and repair [25,26]. Our results showed that the apoptosis rate was lower in the HMB-containing medium (H group) than in the control medium (NC group), indicating that HMB modulates myoblast apoptosis during normal muscle growth and development. Further analyses revealed that DEX induced apoptosis of myoblasts (D group), but HMB treatment (HD group) reversed this effect. Particularly, myoblast apoptosis was comparable between HD and NC groups. This finding demonstrates that DEX induces the apoptosis of myoblasts, but HMB impaired or reversed this effect. In addition, cell apoptosis was not significantly different between myoblasts treated with HMB followed by DEX (HD group) and those treated with HMB and DEX simultaneously (H+D group). The proportional ratio of 6 h blank control cultures to 6 h HMB cultures is insignificant because HMB is both a nutrition and a medication [27]. Although the apoptosis rate was high in the H+D group, the rate was lower than that in the D group, indicating that to a certain extent, HMB and DEX co-treatment still inhibited cell apoptosis. Holeček et al. revealed comparable findings, in which HMB was demonstrated to reduce exercise-induced muscle injury, increase muscle regeneration, and attenuate and prevent sarcopenia in the elderly, promoting muscle growth and power [28,29].

IL-17 is a cytokine produced by T helper 17 (Th17) cells and plays an important role in autoimmune disorders, inflammation, and allergy syndromes [30,31]. IL-17 regulates cartilage formation via the NF-κB pathway [32]. In this study, we found that IL-17 inhibits NF-κB signaling, reducing muscle cell degradation. Further analysis showed that DEX activates NF-κB expression. NF-κB is an important nuclear transcription factor degraded via the ubiquitin-protease pathway [33]. The western blot results revealed that DEX activates NF-κB signaling by phosphorylating NF-κB p65, IKBα, and IKKα proteins, consistent with Jimi et al. findings that IKB and IKK regulate NF-κB activation [32]. Due to proliferation data and the decrease in NF-κB p65, IKBα, and IKKα phosphorylation in the DH group, we speculate that HMB may promote myoblast repair by regulating NF-κB signaling [32].

IL-6 is a key downstream target gene of NF-κB [34]. IL-6 is a soluble pleiotropic cytokine that plays an important role in inflammation, immune responses, and hematopoiesis. IL-6 also mediates tissue injury repair and acute phase response to infection [35]. IL-6 is critical for hematopoiesis in the liver and good health, and dysregulated IL-6 expression can lead to diseases such as rheumatoid arthritis, osteoporosis, and cirrhosis [36]. Dysregulated IL-6 expression can also lead to systemic disorders, slow growth, and even death [37]. The pathological role of IL-6 has been demonstrated in several animal disease models. IL-6 is transiently expressed in response to environmental stressors such as infection and tissue damage [38]. In the present study, IL-6 expression was higher in the D group than in the NC group and DH group. IL-6 expression is primarily activated by TNF-α and IL-1β [39,40]. ELISA analysis revealed that their levels in each group were what we expected, demonstrating that HMB prevents muscle injury via the IL-17/NF-κB pathway.

## 4. Materials and Methods

### 4.1. Cell Isolation and Culture

Two-month-old Tan lamb (Ningxia, China) were anesthetized with 2% lidocaine hydrochloride (4 mg/kg) by intravenous injection, and biceps femoris tissues were harvested. SMSCs were extracted from the tissues using 0.2% collagenase and 0.25% trypsin digestion, cultured, passaged, and cryopreserved. Second passage SMSCs were cultured in complete medium and identified with mouse anti-paired box 7 (*Pax7*) primary antibody (1:200) and fluorescein isothiocyanate (FITC)-labeled goat anti-mouse secondary antibody. When the SMSCs reached 60–70% confluency, the culture medium was replaced with a myoblast differentiation medium, which was changed every 2 days (Table 1). After differentiation, the cells were identified with mouse anti-myogenesis regulatory factor 1 (*MyoD1*; 1:200) and anti-*Desmin* (1:100) primary antibodies, and FITC-labeled goat anti-mouse secondary antibodies. For passaging, myoblasts were treated with 0.25% trypsin for 2 min and mixed with a complete medium to stop the reaction. The digestion step was repeated 1–2 times, and the cells were examined under a microscope. Skeletal muscle cells could adhere firmly to the culture flask, while previously adhered impure cells would float after digestion. The culture flask was vigorously shaken and decanted to remove impure cells.

### 4.2. Construction of Myoblast Injury Model

Glucocorticoid dexamethasone (DEX) can increase protein degradation. Myoblasts were treated with 0.4, 0.6, 0.8, 1.0, or 1.2 mmol/L DEX to induce injury. Pre-experiment showed that 0.8 mmol/L DEX had the best effect on myoblasts, which was primarily marked or characterized by cell apoptosis rate (about 30% injury). HMB concentrations of 0, 0.25, 0.5, 0.75, and 1 μmol/L were applied to the injured muscle cells. Pre-experiments demonstrated that the best effect on myoblasts was at 0.5 μmol/L HMB. Therefore, 6 treatment groups were designed based on this concentration as follows.

NC group: Normal control group cultured in myoblast differentiation medium for 6 h; H group: 12 h of culture in myoblast differentiation medium supplemented with 0.5 μmol/L HMB; D group: 6 h of culture in myoblast differentiation medium supplemented with 0.8 mmol/L DEX; DH group: 6 h of culture in myoblast differentiation medium supplemented with 0.8 mmol/L DEX, followed by 12 h of culture in myoblast differentiation medium supplemented with 0.5 μmol/L HMB; H+D group: 6 h of culture in myoblast differentiation medium supplemented with 0.5 μmol/L HMB and 0.8 mmol/L DEX, followed by 6 h of culture in myoblast differentiation medium supplemented with 0.5 μmol/L HMB; HD group: 12 h of culture in myoblast differentiation medium supplemented with 0.5 μmol/L HMB, followed by 6 h of culture in myoblast differentiation medium supplemented with 0.8 mmol/L DEX. After each group’s course of treatment was complete, samples were taken all at once. In order to maintain consistency, data from 12 h HMB treatments in the H, DH, H+D, and DH groups were used. 

### 4.3. Detection of Cell Apoptosis by Flow Cytometry

Cells were cultured in 60 mm culture flasks under the respective treatment conditions in triplicate. Cells were then collected, centrifuged, and resuspended in PBS (10^5^ cells/mL). The culture medium supernatant was also gathered in order to ensure that all cells were extracted. The cells were incubated with 100 μL 1X binding buffer, 5 μL Annexin V-FITC and 5 μL propidium iodide (PI) at room temperature for 10 min, followed by the addition of 200 μL 1X binding buffer (volume based on cell number). The apoptosis rates were analyzed by flow cytometry.

### 4.4. EdU Assay 

SMSCs were seeded in a 24-well plate to differentiate into myoblasts. Cells were stained using the BeyoClick^TM^ EdU Azide488 test kit (Beyotime, Shanghai, China) according to the manufacturer’s instructions. Three random fields of view were photographed well under a fluorescence microscope (Beyotime, Shanghai, China), and the proportion of EdU-positive cells was calculated (Azide488-positive cells/Hoechst-positive cells). The analysis was performed in triplicate.

### 4.5. ELISA

Briefly, 50 μL of the test samples (final sample concentration was 1/5) was added to the ELISA wells and incubated at 37 °C for 30 min. The plate was washed 5 times, treated with an enzyme-conjugated reagent for 30 min at 37 °C, washed 5 times, and incubated with 50 μL/well chemiluminescence solutions A and B at 37 °C for 10 min in the dark. Stop solution (50 μL/well) was added to each well, and the ODs were measured using a microplate reader within 15 min.

### 4.6. Western Blot

Total proteins were extracted from myoblasts in the NC, D, and DH groups, concentrated in a 5% stacking gel (90 V for 30 min), and separated in 10% separation gel (120 V for 90 min). Protein sample concentration was calculated and standardized. The separated proteins were transferred (220 mA for 1 h) onto a PVDF membrane, blocked with BSA for 1 h at room temperature, and incubated with primary antibodies (anti-NF-κB p65 (CST, D14E12, 1:1000; Cell Signaling Technology, Beverly, MA, USA), IKKα (ab32041, 1:1000; Abcam, Cambridge, UK), IKBα (ab32518, 1:1000; Abcam, Cambridge, UK) and GAPDH (ProProtection, 1:5000; Abcam, Cambridge, UK)) at 4 °C overnight, and thereafter with HRP-conjugated goat anti-rabbit IgG (Zhongshan Bio-tech Co., Ltd., Beijing, China) for 1 h at room temperature, washed 5 times with TBST (5 min/wash), and incubated with chemiluminescence solution for 1 min at room temperature. The PVDF membrane was quickly placed on an X-ray film and exposed in a dark room. The film was developed in a film processor, and the exposure time for band visualization was optimized. For phosphorylation detection, anti-p-NF-κB p65 (ab133462, 1:1000; Abcam, Cambridge, UK), anti-p-IKKα (CST, 16A6, 1:1000; Cell Signaling Technology, Beverly, MA, USA), and anti-p-IKBα (CST 93H1, 1:1000; Cell Signaling Technology, Beverly, MA, USA) primary antibodies were used.

### 4.7. RNA-Seq Analysis

RNA extraction and RNA-seq analysis were performed by majorbio (Shanghai, China), the sequencing platform was illumina novaseq 6000, and the read length was 2 × 150 bp. The raw paired end reads were trimmed and quality controlled by fastp (0.19.5) (https://github.com/OpenGene/fastp) with default parameters, accessed on 31 March 2022 [41]. Then clean reads were separately aligned to reference genome with orientation mode using HISAT2 (2.1.0) (http://ccb.jhu.edu/software/hisat2/index.shtml), accessed on 31 March 2022. Ovis aries was the reference gene source, the reference genome version was GCF 016772045.1, and the reference genome sources were https://www.ncbi.nlm.nih.gov/genome/?term=txid9940 [orgn] software, accessed on 31 March 2022 [42]. The mapped reds of each sample were assembled by StringTie (2.1.2) (https://ccb.jhu.edu/software/stringtie/) in a reference-based approach, accessed on 31 March 2022 [43].

Use DESeq2 software (1.24.0) to perform differential expression analysis between two comparative combinations (three biological replicates for each group). DESeq2 provides statistical procedures for determining differential expression in digital gene expression data using models based on the negative binomial distribution. We used the method of Benjamini and Hochberg to adjust the *p* value to control the false discovery rate. The reads count and RNA quality information of each sample are listed in Table A1. The selection criteria for differentially expressed genes (DEGs) are padj ≤ 0.05, and |log2foldchange| ≥ 1. In addition, functional-enrichment analysis including GO (Gene Ontology, http://www.geneontology.org), accessed on 31 March 2022 and KEGG (Kyoto Encyclopedia of Genes and Genomes, http://www.genome.jp/kegg/), accessed on 31 March 2022, were performed to identify which DEGs were significantly enriched in GO terms and metabolic pathways at *p*-adjust ≤ 0.05 compared with the whole-transcriptome background. GO functional enrichment and KEGG pathway analysis were carried out by Goatools (https://github.com/tanghaibao/Goatools), accessed on 31 March 2022 and KOBAS (http://kobas.cbi.pku.edu.cn/home.do), accessed on 31 March 2022 [44].

### 4.8. Statistical Analysis

Data were analyzed using the 2019 Excel package (Microsoft, Redmond, WA, USA) and SPSS 27.0 (IBM, Armonk, NY, USA). Differences among groups were compared by the Duncan test. Bar graphs were generated using GraphPad Prism software, version 8.0 (GraphPad Software, San Diego, CA, USA). * *p* < 0.05 was considered statistically significant and ** *p* < 0.01 indicated very high statistical significance.

## 5. Conclusions

In this study, we successfully extracted Tan sheep SMSCs and assessed the apoptosis and proliferation of these cells following an injury. Our results showed that HMB promotes the repair of muscle cell injury by inhibiting IL-17 secretion, which in turn inhibits NF-κB signaling, and, consequently, the expression of IL-6, TNF-α, and IL-1β. The discoveries of this study provide new insights to improve the meat quality of Tan sheep.

## Figures and Tables

**Table 1:** Culture medium ingredients.

Medium	Ingredients
Complete medium	data High-glucose medium + 10%FBS + 1% penicillin/streptomycin (P/S)
Myoblast Differentiation medium	data High-glucose medium + 2%HS
Storage and transport	PBS + 5%P/S

## Data Availability

Not applicable. And data presented in this study are available on request from the corresponding author. RNAseq raw data was available at NCBI on GenBank and SRA using the following BioProject IDs: PRJNA905121.

## Appendix B

**Table A1:** Information of RNA-seq samples.

Sample	Raw Reads	Raw Bases	Clean Reads	Clean Bases	Error Rate (%)	Q20(%)	Q30(%)	GC Content
								(%)
NC1	6,100,7804	9,212,178,404	59,795,486	8,641,876,217	0.0252	97.86	94	53.63
NC2	5,404,5836	8,160,921,236	52,765,256	7,614,406,901	0.0256	97.69	93.62	53.8
NC3	5,465,8296	8,253,402,696	53,098,864	7,627,471,914	0.0254	97.77	93.87	53.77
H1	58,538,782	8,839,356,082	57,553,362	8,395,202,659	0.0251	97.93	94.16	53.55
H2	52,922,990	7,991,371,490	51,795,790	7,545,225,170	0.0257	97.66	93.54	53.5
H3	49,916,272	7,537,357,072	48,886,386	7,108,693,973	0.0249	97.98	94.32	53.38
D1	54,033,588	8,159,071,788	52,352,756	7,584,780,012	0.0253	97.8	93.95	53.79
D2	62,382,402	9,419,742,702	61,289,950	8,929,993,888	0.0252	97.88	94.03	53.47
D3	52,905,810	7,988,777,310	51,661,206	7,559,307,614	0.0253	97.8	93.91	53.46
HD1	54,294,954	8,198,538,054	53,026,950	7,734,884,116	0.0255	97.75	93.78	53.74
HD2	52,512,512	7,929,389,312	51,447,990	7,544,672,078	0.0251	97.93	94.18	53.33
HD3	51934726	7,842,143,626	50,781,802	7,445,053,103	0.025	97.93	94.21	53.39
D+H1	54,951,310	8,297,647,810	53,563,784	7,798,454,682	0.0255	97.72	93.71	53.69
D+H2	52,651,856	7,950,430,256	50,706,446	7,391,244,697	0.0256	97.67	93.66	53.6
D+H3	50,439,524	7,616,368,124	48,796,802	7,106,788,337	0.0254	97.8	93.9	53.71
DH1	57,570,642	8,693,166,942	56,450,908	8,226,601,480	0.025	97.94	94.22	53.38
DH2	57,144,710	8,628,851,210	55,878,600	8,101,309,031	0.0251	97.9	94.12	53.5
DH3	46,672,916	7,047,610,316	45,545,154	6,640,024,249	0.0252	97.85	94.06	53.59
